# *Koenigia medogensis* (Polygonaceae: Persicarieae), a Distinct New Species From Xizang, Southwestern China

**DOI:** 10.1002/ece3.72089

**Published:** 2025-08-31

**Authors:** Xiao‐Ting Xie, Yi‐Ming Wei, Yong‐Jun Chen, Dian‐Xiang Zhang, Jian‐Yong Shen, Yao‐Wu Xing, Bo Li

**Affiliations:** ^1^ Center for Integrative Conservation, Xishuangbanna Tropical Botanical Garden, Chinese Academy of Sciences Mengla China; ^2^ College of Life Sciences, University of Chinese Academy of Sciences Beijing China; ^3^ College of Agronomy, Jiangxi Agricultural University Nanchang China; ^4^ Center for Gardening and Horticulture, Xishuangbanna Tropical Botanical Garden, Chinese Academy of Sciences Mengla China; ^5^ Yunnan Key Laboratory of Forest Ecosystem Stability and Global Change, Xishuangbanna Tropical Botanical Garden Chinese Academy of Sciences Mengla China

**Keywords:** micromorphology, palynology, *Persicaria*, phylogeny, Polygonoideae

## Abstract

*Koenigia medogensis*, a distinctive new species discovered in Medog County, southeastern Xizang, China, is here described and illustrated. The generic placement of this species was validated through integrated morphological and palynological observations, as well as molecular phylogenetic analyses using three cpDNA markers (*matK*, *rbcL*, and *trnL‐F*). Within *Koenigia*, *K. medogensis* is most closely related to *K. mollis* but differs significantly in growth habit, leaf shape, inflorescence structure, and achene micromorphology. Crucially, *K. medogensis* possesses a unique vegetative reproductive strategy, viz. the production of bulbils at both stolon apices and inflorescence apex. This dual‐bulbil trait is exceptionally rare within Polygonaceae and likely represents an evolutionary adaptation to the hyperhumid monsoon environment of Medog County as a reproductive assurance strategy.

## Graphical abstract

In this study, we describe and illustrate a distinctive new species, Koenigia medogensis, discovered in southeastern Xizang of China. We employed an integrative approach combining morphological, palynological, and molecular phylogenetic data to validate its taxonomic placement within Koenigia.

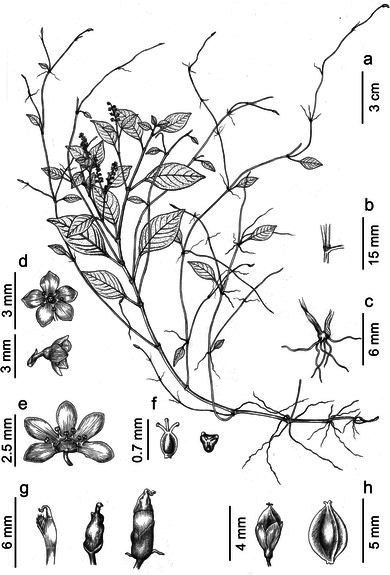

## 1 Introduction

The genus *Koenigia* L. was originally established by Linnaeus (1767) based on a sole species, *K. islandica* L., which is characterized by highly reduced floral features, including three perianths and three stamens alternating with the perianths. For over a century, it has been widely accepted as a monotypic genus due to its unique morphological traits (e.g., Ledebour 1850; Steward 1930; Losina‐Losinskaja 1936; Roberty and Vautier 1964; Tutin et al. 1991; Li et al. 1998, 2003). However, Hedberg (1946) discovered that the characteristic pollen type found in *K. islandica* , that is the outer surface of the pollen wall covering spines, was shared by several species that had traditionally been classified within *Polygonum* sect. *Aconogonon* Meisn. (e.g., *P. nummulariifolium* Meisn. and *P. forrestii* Diels) and sect. *Cephalophilon* Meisn. (e.g., *P. delicatulum* Meisn., *P. cyanandrum* Diels, *P. filicaule* Wall. ex Meisn., and *P. hubertii* Lingelsh.). Thus, the genus was first expanded to incorporate these species (Hedberg 1946). This revised circumscription gained support from subsequent morphological, anatomical, and cytological analyses (Marek 1954, 1958; Haraldson 1978; Ronse Decraene and Akeroyd 1988). Subsequently, Hedberg (1997) conducted a comprehensive taxonomic revision of the *Koenigia* and formally recognized six species and one subspecies, viz. *K. islandica* , *K. nepalensis*, D.Don, *K. nummularifolia* (Meisn.) Měsíček & Soják, *K. pilosa* Maxim., *K. forrestii* (Diels) Měsíček & Soják, *K. delicatula*, (Meisn.) H.Hara subsp. *delicatula*, and *K. delicatula* subsp. *relicta* Hedberg. With the advancement of molecular phylogenetic studies, Schuster et al. (2015) proposed to further expand the circumscription of *Koenigia* to include all species of *Aconogonon* to achieve monophyly, as neither genus was monophyletic under their previous circumscriptions. This taxonomic treatment was supported by several morphological, anatomical, and palynological evidences, such as disc‐shaped nectaries with basally fused inner stamens (Ronse Decraene and Akeroyd 1988; Hong 1992), unicellular trichomes (Haraldson 1978), and pollen exine sculpture patterns (Hong and Hedberg 1990). The redefined *Koenigia* now encompasses approximately 60 species distributed from Arctic tundra to temperate alpine ecosystems throughout the Northern Hemisphere and constitutes the tribe Persicarieae of the subfamily Polygonoideae, along with *Bistorta* (L.) Scop. and *Persicaria* Mill. (Schuster et al. 2015).

Medog County (Tibetan: མེ་ཏོག་རྫོང་།; Chinese: 墨脱县) is located in the southeastern Xizang Autonomous Region, southwestern China, occupying the southern slope of the Himalayas and the lowest area in the Qinghai‐Tibet Plateau, which is the highest and most extensive plateau in the world (Liu 2019). Due to the heavy precipitation caused by the South Asian monsoon, Medog County is known for its unique subtropical humid climate and the presence of the northernmost tropical monsoon rainforests in China, a rarity on the Qinghai‐Tibet Plateau (Sun and Zhou 1996; Tang 2015; Hu et al. 2020). This region exhibits a complex topography characterized by towering mountain ranges, expansive alpine meadows, and distinctive glacial landforms, forming a critical biodiversity hotspot within the Sino‐Himalayan flora (Ding et al. 2022). Recent advancements in transportation infrastructure have enabled extensive biodiversity surveys and specimen collection efforts by Chinese research institutions (Liu et al. 2020). These initiatives have led to the discovery and formal description of multiple new taxa (Li et al. 2018; Wang et al. 2019; Ya et al. 2019; Liu et al. 2020; Luo et al. 2020; Fu et al. 2021; Ding et al. 2022). However, significant knowledge gaps remain regarding the region's flora, with many species yet to be documented (Ding et al. 2022; Liu et al. 2020).

During repeated field surveys from 2017 to 2024 in Medog County, southeastern Xizang, China, a distinctive Polygonaceae species was consistently collected near Lage village of Dexing Town. The plant exhibits dehiscent membranous tubular ocreae, terminal umbellate‐racemose inflorescences, trigonous achenes exceeding persistent perianth, and extremely short styles, suggesting an initial generic affinity with *Koenigia*. Subsequent molecular phylogenetic analyses using three chloroplast markers (*matK*, *rbcL*, and *trnL‐F*) confirmed its placement within *Koenigia*. However, detailed examination revealed its distinction from all known congeners by possessing bulbils at both stolon apices and basal inflorescence axes. Such a vegetative reproductive structure is exceptionally rare in Polygonaceae, previously documented only in *Bistorta vivipara* (L.) Gray (Diggle 1997; Diggle et al. 2002). Palynological and micromorphological observations further supported its taxonomic status as a novel species. Therefore, we herein formally describe and report this new species as *Koenigia medogensis* Bo Li.

## 2 Materials and Methods

### 2.1 Morphological Description

Field surveys were conducted across Medog County from 2017 to 2024. Herbarium specimens of *Koenigia* deposited at CDBI, HITBC, IBSC, KUN, HNWP, NAS, PE, QFNU, SZ, WUK, and XZ (acronyms follow Thiers, 2025+) were examined. Morphological observations and descriptions of the putative new species are based on living plants observed in situ as well as dried specimens collected from the type locality. Quantitative traits (e.g., plant height, leaf size, and floral organ dimensions) were measured using a steel ruler (0.5 mm precision) and digital calipers (±0.01 mm). Achene and bulbil morphology were photographed using SmartZoom5 stereomicroscopy (SM) (Carl Zeiss, Germany) with digital imaging.

### 2.2 Micromorphological Observation

Pollen morphology and achene surface sculpture of the putative new species were observed using scanning electron microscopy (SEM). Mature achenes and well‐developed unopened flowers were removed from dry specimens. All samples were initially cleaned using ultrasonic treatment in 95% ethanol. Subsequently, the pericarp was carefully peeled off, and pollen grains were extracted from the anthers, placed onto conductive adhesive, and mounted onto copper stubs. The samples were coated using a Q150RS ion sputter coater (Quorum Tech. Ltd., UK). Observations and microphotography were conducted under SmartZoom5 SM and EVOLS10 SEM (Carl Zeiss, Germany) at a voltage of 8 kV. Terminology for achenes and pollen grains followed Kong and Hong (2018) and Kong et al. (2021), respectively. Voucher specimens are deposited in the herbarium of the Xishuangbanna Tropical Botanical Garden (HITBC), Chinese Academy of Sciences.

### 2.3 Taxon Sampling, Choice of Markers, and Datasets

To verify the generic placement of the putative new species, we conducted phylogenetic analyses using a concatenated dataset of three chloroplast DNA markers (*matK*, *rbcL*, and *trnL‐F*), which have been widely adopted for resolving relationships within *Koenigia* and Polygonaceae (e.g., Lamb‐Frye and Kron 2003; Burke et al. 2010; Fan et al. 2013; Schuster et al. 2015). The ingroup taxon sampling focused on Polygonoideae, encompassing all major clades of the subfamily, with specific emphasis on dense sampling of *Koenigia*. A total of 19 genera and 60 species were sampled, including *Antigonon leptopus* Hook. & Arn. of Eriogonoideae as an outgroup. GenBank accession numbers are listed in Table S1.

### 2.4 DNA Extraction, Amplification, and Sequencing

DNA was extracted from silica gel‐dried leaves according to the manufacturer's instructions for the DNeasy Plant Mini Kit (Qiagen, Valencia, CA, USA). PCR reactions and amplification procedures were performed following the method described by Schuster et al. (2011). The PCR products were purified using a PCR Product Purification Kit (Shanghai Sangon Biotech Ltd., China) and were subsequently used for sequencing. Electropherograms were assembled, and consensus sequences were generated with the Geneious Prime 2025.0.2 platform.

### 2.5 Phylogenetic Analyses

MAFFT v.7.526 (Katoh and Standley 2013) was employed to conduct sequence alignment for each separate matrix with default parameters and further concatenate them into a combined dataset. Phylogenetic relationships were inferred using Maximum Likelihood (ML) and Bayesian Inference (BI) analyses. ML analysis was conducted in Stamatakis‐HPC2 v.8.2.11 (Stamatakis 2014) under the GTRGAMMA model, with 1000 bootstrap replicates to estimate Maximum Likelihood Bootstrap Support (MLBS) values for branches/nodes. BI analysis was performed using MrBayes v.3.2.7 (Ronquist et al. 2012). Bayesian Information Criterion (BIC) was applied to determine the best‐fit DNA substitution model for each dataset using jModelTest 2 (Darriba et al. 2012). Markov chain Monte Carlo (MCMC) simulations were run for 3,000,000 generations, with trees sampled every 1000 generations. The average standard deviation of split frequencies for each dataset was below 0.01, and the potential scale reduction factor (PSRF) for convergence diagnostics was 1.00, indicating that the number of generations was sufficient. The first 50% of the sampled trees were discarded as burn‐in, and the remaining trees were used to construct a majority‐rule consensus tree. Bayesian posterior probability values (BI‐PP) ≥ 0.95 were considered to indicate strong support. Tree visualization was performed using the online tool iTOL (https://itol.embl.de/) (Letunic and Bork 2024).

## 3 Results

### 3.1 Morphological Comparisons

**FIGURE 1:**
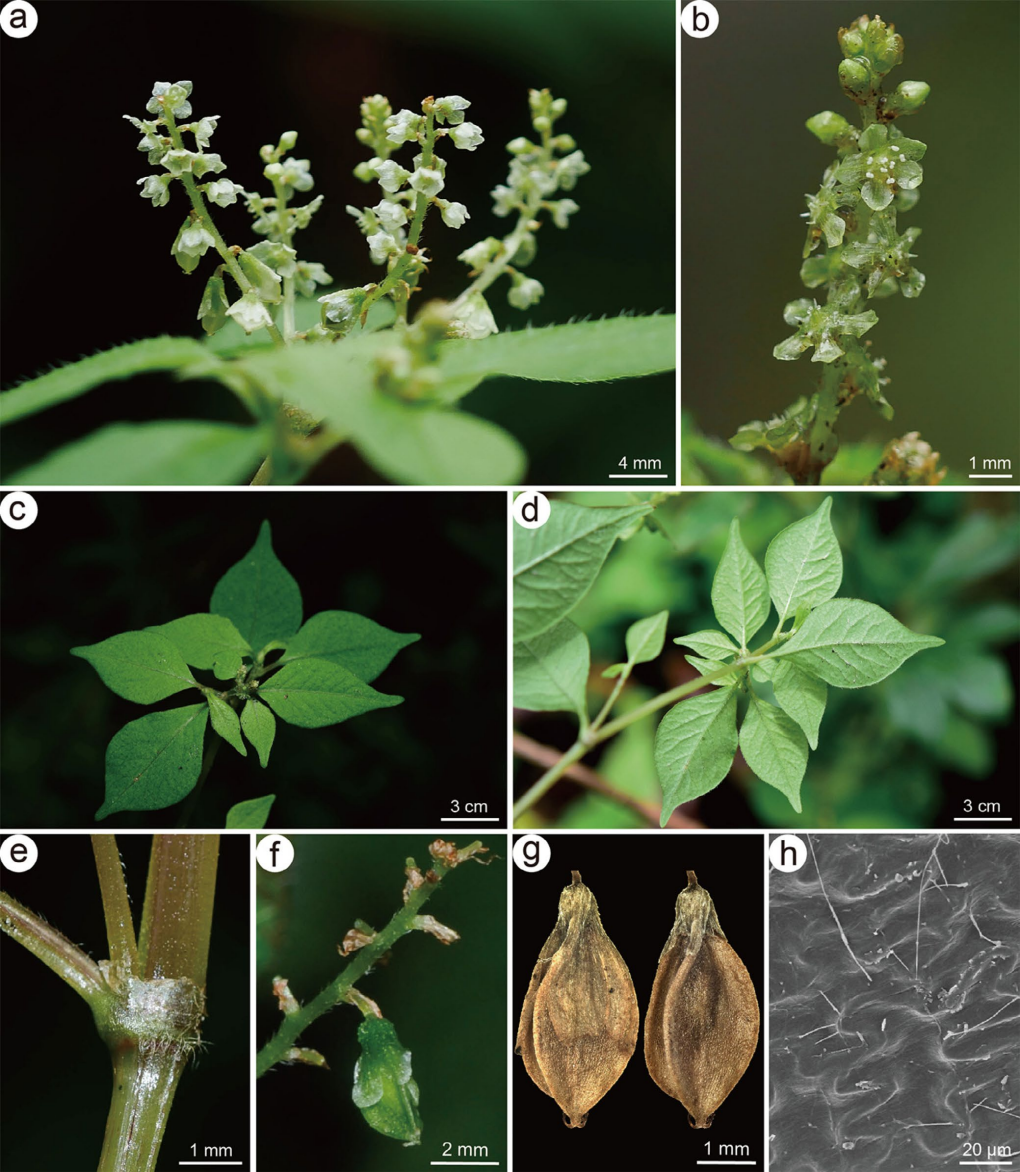
Partial morphological characteristics of *Koenigia medogensis*. (a) inflorescence, (b) flowers, (c, d) leaves, (e) ocrea, (f, g) persistent pedicel and trigonous achene, (h) achene surface sculpture (a, b were photographed by Hai‐Lei Zheng, c–e by Hong Jiang, f by Bing Liu, and g, h by Xiao‐Ting Xie).

**FIGURE 2:**
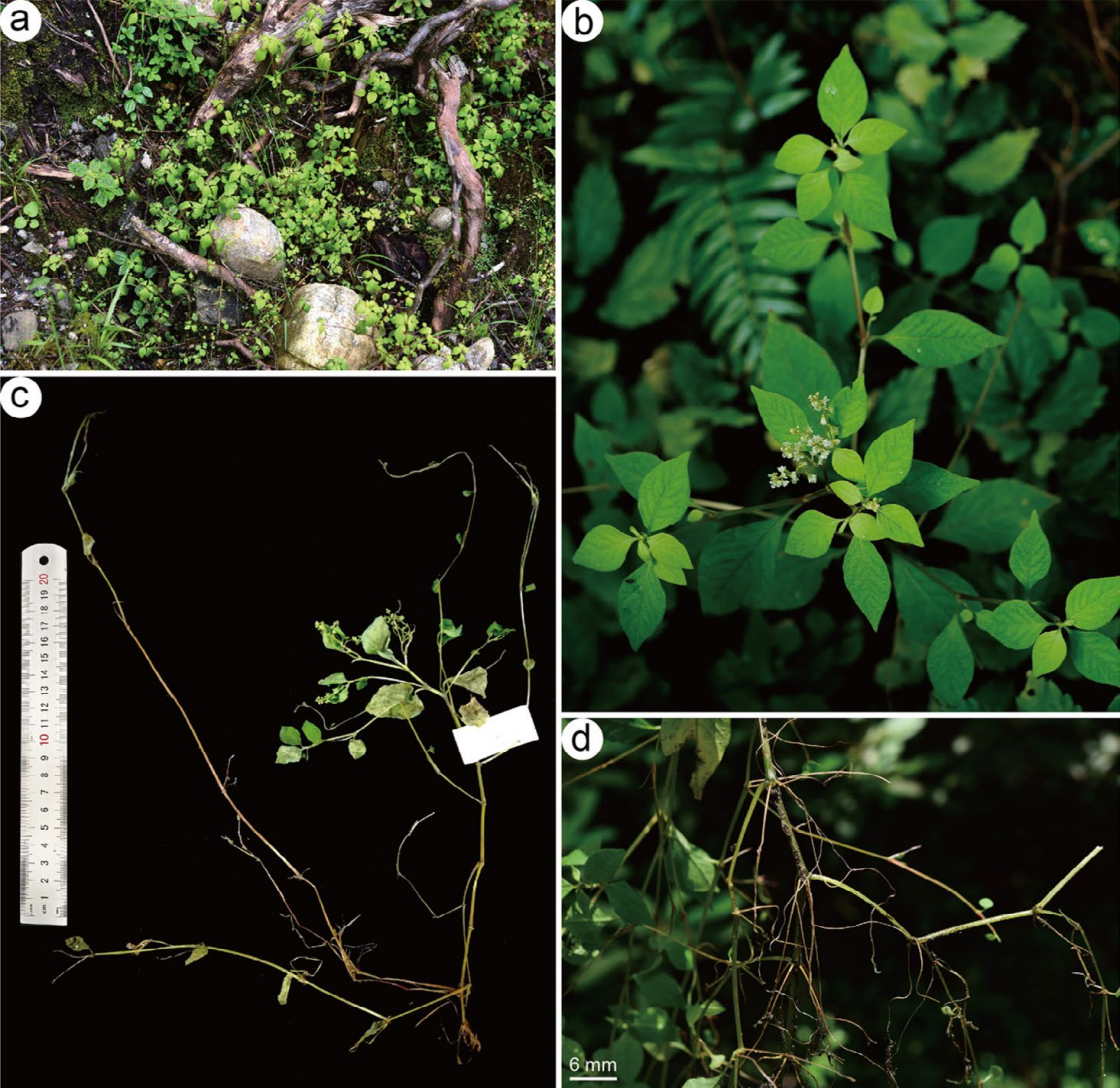
Morphology of *Koenigia medogensis* Bo Li, sp. nov.: (a) habitat, (b, c) habit, and (d) abundant roots at lower nodes (a, b, and d were photographed by Hong Jiang, and c by Hai‐Lei Zheng).

**FIGURE 3:**
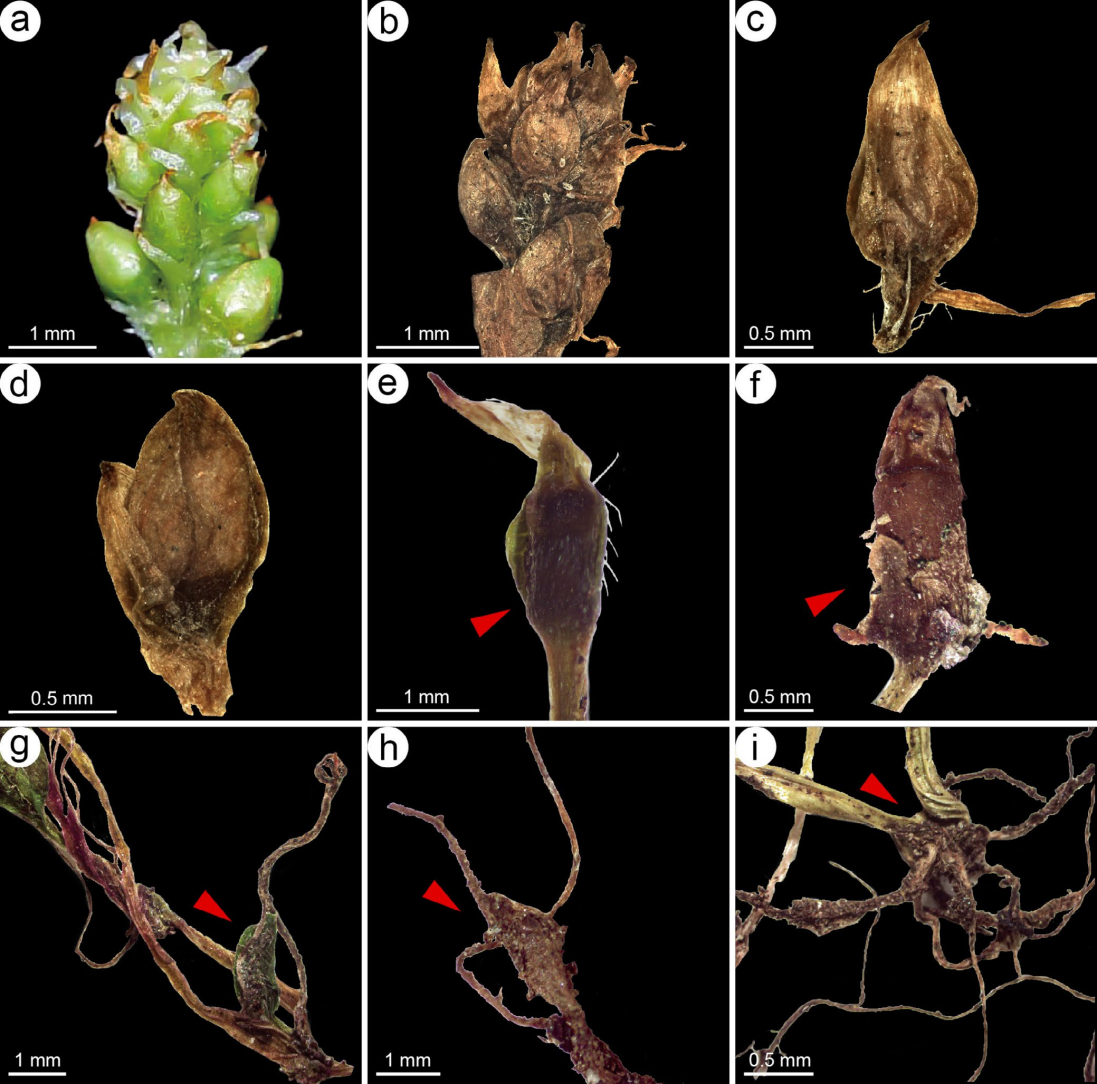
Bulbil structure of *Koenigia medogensis*. (a–d) bulbils at the inflorescence apex, (e–i) root formation in bulbils (shown by red arrows) at the stolon tip (a was photographed by Hong Jiang, b–i by Xiao‐Ting Xie).

In overall morphology, *K. medogensis* is most similar to *K. filicaulis* (Wall. ex Meisn. T.M.Schust. & Reveal) and *K. pilosa* in having thin, ascending to reclining stems with many branches from the base. However, it differs distinctly in inflorescence type, achene morphology, and the relationships between achene and perianth. *K. medogensis* is a perennial herb that shares the same growth habit as *K. forrestii*, *K. nummularifolia*, and *K. hookeri* (Meisn.) T.M.Schust. & Reveal. However, these species are readily distinguished by differences in indumentum type, leaf shape and size, inflorescence type, and perianth length. Although *K. medogensis* and *K. mollis* (D.Don) T.M.Schust. & Reveal exhibit striking similarities not found in other *Koenigia* species, both possess densely pubescent inflorescence axes along with small flowers that are fully spreading (Figure 1a,b). Their inflorescence type differs markedly, viz. *K. mollis* produces large paniculate inflorescences, while *K. medogensis* develops umbellate‐racemose inflorescences (Figure 1a; Table 1). Besides, *K. medogensis* possesses stolons (Figure 2d) and terminal bulbils (Figure 3), key characteristics to distinguish it from other *Koenigia* species.

**TABLE 1:** Morphological comparison of *K. medogensis* with other *Koenigia* species.

		*K. medogensis*	*K. mollis*	*K. islandica*	*K. forrestii*	*K. nummularifolia*	*K. hookeri*	*K. filicaulis*	*K. pilosa*
Growth Habit		Herbs perennial	Subshrubs	Herbs annual	Herbs perennial	Herbs perennial	Perennial herb	Herbs annual	Herbs annual
Stem	Morphology	Ascending or prostrate, tufted	Erect, much branched	Dwarf; stems erect; slender	Stems creeping, tufted; branches erect	Dwarf, stems creeping	Erect, unbranched	Ascending or reclining, tufted	Erect, ascending or reclining; slender, angulate, branched
	Indumentum	Sparsely pubescent or glabrous	Hirsute, retrorsely hirsute or glabrous	Glabrous	Villous	Smooth or sparsely pubescent	Sparsely hirsute	Sparsely strigose, with reflexed hairs below nodes	Pilose or glabrous
Leaf	Shape	Ovate or ovate‐lanceolate, apex acute or shortly acuminate	Elliptic or elliptic‐lanceolate, apex acuminate	Broadly elliptic, obovate, or nearly orbicular, apex obtuse	Suborbicular or reniform, apex rounded	Orbicular to reniform, apex obtuse	Basal leaves narrowly elliptic or spatulate, apex rounded	Blade ovate or lanceolate‐ovate, apex acute	Broadly ovate, apex obtuse
	Indumentum	Sparsely pubescent on both surfaces	Abaxially sericeous, adaxially sparsely sericeous	Glabrous on both surfaces	Both surfaces long pilose or nearly glabrous	Abaxially pilose, adaxially glabrous	Both surfaces hirsute, abaxially densely hirsute along midvein	Both surfaces densely or sparsely strigose	Both surfaces pilose
	Size (cm)	1.0–3.5 × 0.5–2.2	10.0–20.0 × 3.0–6.0	3.0–6.0 × 2.0–4.0	1.0–4.0	0.5–2.0	5.0–10.0 × 1.5–3	1.0–3.5 × 0.5–1.3	0.8–2.5 × 0.5–1.0
	Base	Cuneate, slightly narrow at base	Cuneate	Broadly cuneate	Cordate	Cordate	Narrowly cuneate	Cuneate	Base broadly cuneate or subtruncate
Ocrea	Shape	Tubular, bifid	Tubular, oblique	Tubular	Tubular, oblique	Tubular, oblique	Tubular, apically oblique	Funnel‐shaped	Tubular, distinctly bilobed at apex
Inflorescence	Type and Form	Racemose inflorescence, umbrella‐shaped, flowers spreading	Inflorescence spreading, paniculate, large	Flowers axillary, fascicled	Inflorescence terminal, corymbose‐cymose	Inflorescence terminal, corymbose, dense	Inflorescence paniculate, terminal	Inflorescence axillary or terminal, capitate	Inflorescence terminal or axillary, capitate
Tepal	Perianth Color	White or greenish	White	Greenish	White or yellowish	White	Purple‐red	White or pinkish	White or greenish
	Length (mm)	1.0–2.5	1.5–2.0	1.0	4–5, unequal	2–3	2.0–3.0	1.0–2.4	1.0–1.6
	Number	5‐parted	5‐parted	5‐parted	5 (or 4)‐parted	5‐parted	5‐parted	5‐parted	4‐parted
Stamen Number		8	8	3	6–8	8	8	3–4	2–5
Achene	Shape	Ovoid, distinct trigonous shape	Ovoid, distinct trigonous shape	Oblong‐ovoid, biconvex	Narrowly ellipsoid, trigonous, narrow at base	Broadly ovoid, biconvex	Broadly ovoid, trigonous	Ovoid, distinctly 3‐angled, sides concave	Ovoid, 3‐angled
	Relationship with perianth	Exceeding persistent fleshy perianth	Slightly exceeding perianth	Slightly exceeding persistent perianth	Included in persistent perianth	Included in persistent perianth	Slightly exceeding persistent perianth	Slightly exceeding persistent perianth	Included in persistent perianth
	Surface	Smooth, translucent to slightly shiny	Shiny	Granular, dull	Dull	Slightly shiny	Shiny	Shiny	Slightly shiny
Adventitious Roots		Present at internodes	Absent	Absent	Absent	Present at internodes	Absent	Absent	Absent
Bulbil		Apex	Absent	Absent	Absent	Absent	Absent	Absent	Absent

### 3.2 Pollen Micromorphology

**FIGURE 4:**
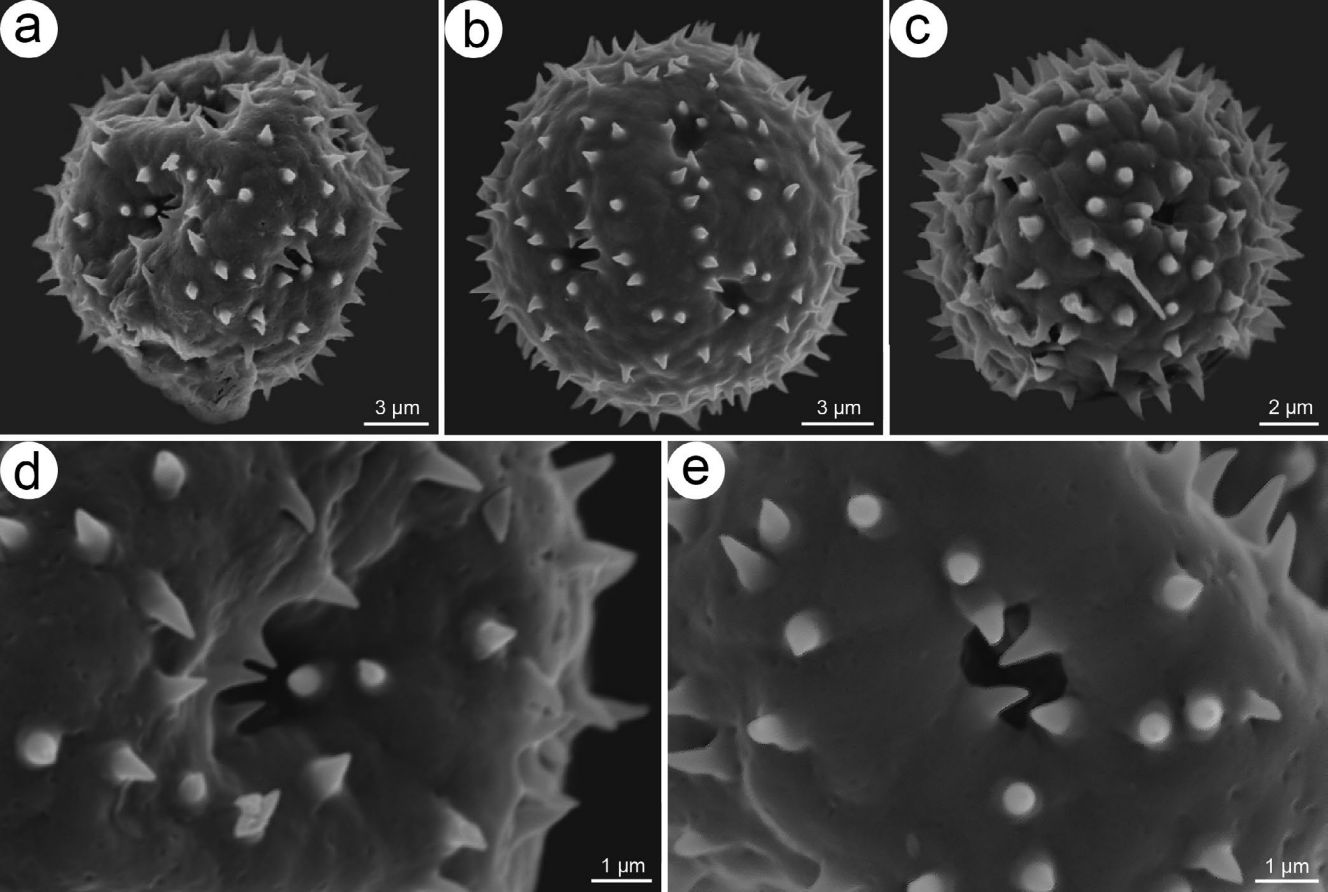
SEM micrographs of pollen grains of *Koenigia medogensis*. (a) polar view, (b, c) equatorial view, and (d, e) exine surface and aperture.

Pollen grains of *K. medogensis* are spheroidal (11.5–20.0 μm in diameter), with a polygonal to nearly circular outline, 12(−15) pantoporate apertures (circular pores, 0.9–2.0 μm in diameter), and distinctly spinulose exine ornamentation (spinules 0.2–1.8 μm long) (Figure 4).

### 3.3 Achene Morphology

The achenes of *K. medogensis* were smooth, distinctly sharp‐triangular, with acutely ridged angles and a color ranging from yellowish‐brown to brown. Additionally, the achene surface was irregular with anticlinal cell walls, exhibiting a wavy structure arranged in a jigsaw‐like pattern (Figure 1g,h).

### 3.4 Dataset Characteristics

The combined dataset has 60 aligned sequences and comprises 7144 characters (2670 bp for *matK*, 2961 bp for *rbcL*, and 1513 bp for *trnL‐F*), of which 2938 are variable (41.13%) and 1793 are parsimony‐informative (25.10%), as shown in Table S2.

### 3.5 Molecular Phylogeny

**FIGURE 5:**
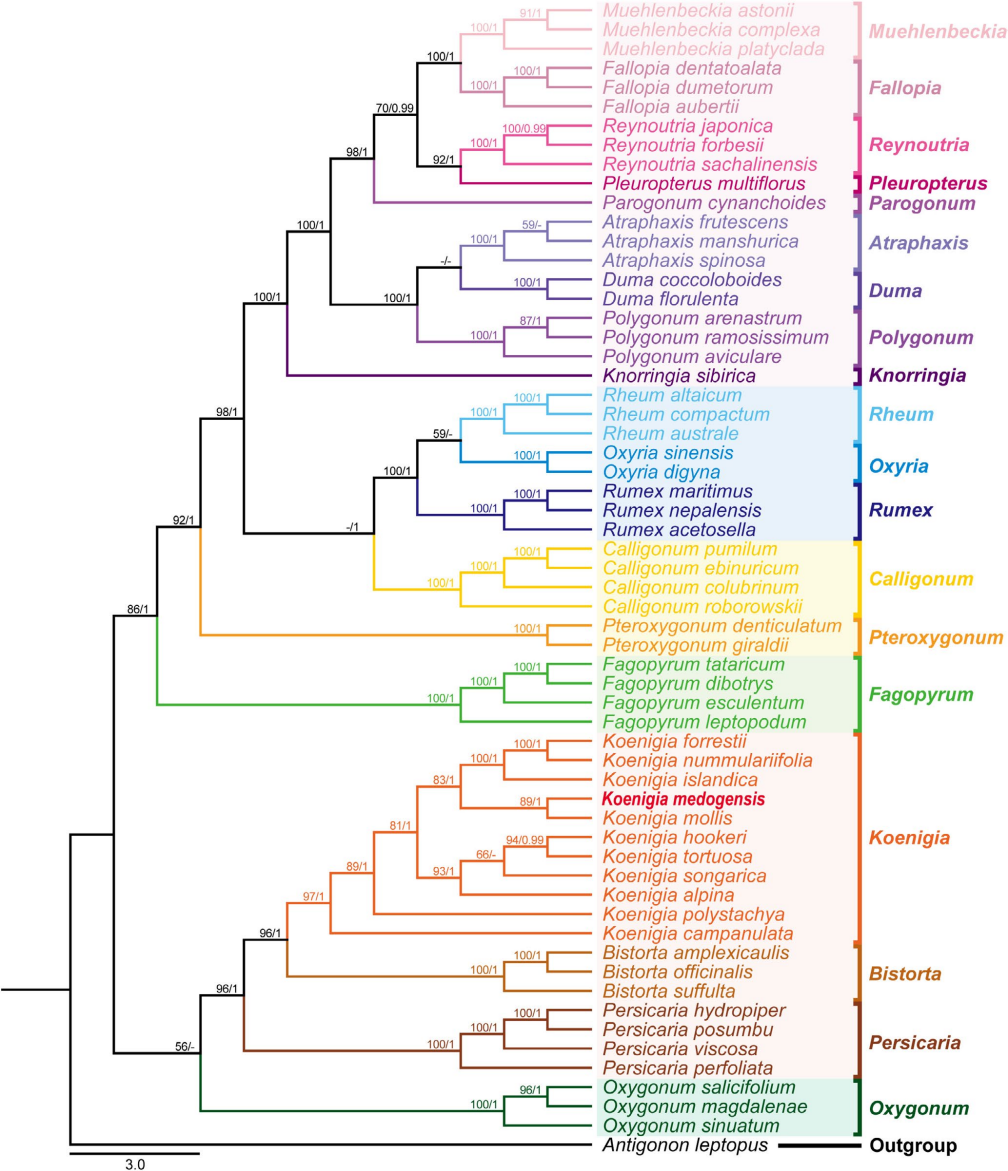
ML and Bayesian phylogenetic tree based on the combined cpDNA (*matK*, *rbcL*, and *trnL‐F*) dataset of the 60 accessions representing 19 genera of Polygonoideae. Support values > 50% ML‐BS or 0.90 BI‐PP are displayed above the branches, respectively, while a dash (−) indicates ML‐BS < 50% or BI‐PP < 0.90. New species are shown in red and bold.

The ML and BI analyses based on our dataset generated nearly identical topologies (Figure 5, Figure S1). Therefore, only the ML tree is presented with the Bootstrap values (ML‐BS) and BI‐PP marked above each branch, respectively (Figure 5). In both of the analyses, *K. medogensis* is placed within *Koenigia* and grouped with *K. mollis* as a highly supported clade (Figure 5; ML‐BS = 89%, BI‐PP = 1.00).

## 4 Discussion

### 4.1 The Distinctive Morphology of *K. medogensis*

The taxonomic history of *Koenigia* has undergone a significant expansion from a monotypic to a polytypic genus. This expansion resulted from the incorporation of several species previously classified under *Persicaria* and *Aconogonon* (Hedberg 1946; Schuster et al. 2015), which has markedly enhanced the species diversity in *Koenigia*. The genus is now characterized by substantial variation in growth forms, inflorescence types, and reproductive strategies. The growth forms are diversified, such as small annual herbs (e.g., *K. islandica* , *K. filicaulis*), perennial herbs (e.g., *K. forrestii*, *K. nummularifolia*, *K. hookeri*), and semi‐shrubs (e.g., *K. mollis* , *K. tortuosa* (D.Don) T.M.Schust. & Reveal). Additionally, inflorescence types in *Koenigia* vary considerably, including large panicle‐like inflorescences, as in *K. campanulata* (Hook.f.) T.M.Schust. & Reveal and *K. polystachya* (Wall. ex Meisn.) T.M.Schust. & Reveal; cymose inflorescences, as in *K. forrestii* and *K. nummularifolia*; and capitate inflorescences, as in *K. delicatula* , *K. pilosa* , and *K. filicaulis*. Furthermore, vegetative reproduction strategies were also developed in some species, exemplified by the presence of adventitious roots developing at internodes in *K. nummularifolia* (https://www.iplant.cn/foc).

Although *Koenigia* exhibits remarkable morphological diversity, the newly discovered species in this study possesses bulbils at both the apex of the inflorescence and the tip of the stolon (Figure 3), which have not been previously reported in *Koenigia*. In the family Polygonaceae, *B. vivipara* is known to produce similar bulbils, and studies have suggested that this trait facilitates adaptation to alpine environments by increasing clonal propagules (Bauert 1993; Fan and Yang 2009). Bulbil formation is also found in various other plants, including *Lilium* L. (Liliaceae), *Pinellia* Ten., *Amorphophallus* Blume, and *Alocasia* (Schott) G.Don (Araceae), as well as *Allium* L. (Amaryllidaceae) (Hao et al. 2024). These asexual structures are widely considered adaptations to harsh environmental conditions (Ai et al. 2020; Shu et al. 2024). Therefore, we hypothesize that the development of bulbils in this new species may represent an evolutionary adaptation to the hyperhumid monsoon environment of Medog County as a reproductive assurance strategy. This characteristic enriches the reproductive strategy diversity of the genus and provides new insights into its evolutionary adaptations to alpine environments.

### 4.2 The Taxonomic Significance of Pollen Micromorphology in *K. medogensis*

Pollen micromorphology, including size, aperture characteristics, and exine ornamentation, holds significant taxonomic value in the phylogenetic studies of Polygonaceae (Ayodele 2005; Hou 2006; Paul and Chowdhury 2020). Notably, in the early classification of *Koenigia* species, pollen morphology served as a primary basis for taxonomic delimitation (Hedberg 1946; Hong and Hedberg 1990; Nowicke and Skvarla 1977). Based on aperture types, Zhou et al. (2004) classified *Koenigia* pollen into three distinct types: *Delicatulum‐type* with 7(−8) equatorial colpi, *Forrestii‐type* with 12 scattered colpi, and *Koenigia‐type* with 12(−15) or 20(−30) scattered pores. In this study, the newly discovered species was found to possess distinct scattered pores, confirming that its pollen type corresponds to the typical *Koenigia*‐type (Figure 4). Furthermore, we found that this new species shares morphological similarities with other *Koenigia*‐type taxa (e.g., *K. islandica* , *K. nepalensis*, *K. pilosa* , *K. pilosa* var. *hubertii* (Lingelsh.) T.M.Schust. & Reveal, *K. fertilis* Maxim., and *K. cyanandra* (Diels) Měsíček & Soják), specifically in possessing slender stems as a common trait. However, differences in leaf indumentum, inflorescence type and morphology, and achene morphology were observed, which further provide diagnostic characters supporting the recognition of this new species.

Based on palynogeographic evidence, it can be inferred that southwestern China alpines may represent the diversification center of *Koenigia* and potentially its place of origin (Zhou et al. 2004). These unique pollen morphological characteristics not only provide crucial taxonomic evidence for distinguishing *Koenigia* from other genera but also reflect the evolutionary relationships within the genus in a phylogenetic context.

### 4.3 The Taxonomic Significance of Achene Micromorphology in *K. medogensis*

During the process of plant identification, fruits and seeds serve as essential diagnostic traits, while surface sculpturing patterns are widely recognized for their systematic significance (Roth 1977; Olowokudejo 1985; Barthlott 1990; Zhang et al. 2005). The morphology of achenes has been extensively investigated in the Polygonaceae (Martin 1954; Oh and Hong 1999; Ayodele and Zhou 2010; Ghimire et al. 2016; Kanwal et al. 2016). Both micromorphological and anatomical features have been recognized as valuable taxonomic characters at both specific and generic levels (Ronse Decraene et al. 2000).

The micromorphological characteristics of the achene micromorphology in *Koenigia* have been reported to exhibit considerable variation and diversity (Kong et al. 2018; Kong and Hong 2018), while the achene micromorphology of the same species from different geographical regions has been found to remain relatively stable (Chen and Zhou 2012). Therefore, achene micromorphology holds potential taxonomic value for species delimitation and identification in *Koenigia*. Kong and Hong (2018) classified achene surfaces into four types: smooth (Type I), tubercled (Type II), small pitted (Type III), and irregularly ridged (Type IV). The newly described species, along with *K. delicatula* , *K. filicaulis*, *K. pilosa* , and *K. islandica*, were classified as Type I (Figure 1h). Nevertheless, distinct morphological differences persist in achene shape, the relationship with the perianth, and surface ornamentation, for example, in comparison with *K. islandica* (Table 1).

### 4.4 Phylogenetic Position of *K. medogensis*

The phylogenetic tree constructed based on the integrated datasets of *matK*, *rbcL*, and *trnL‐F* has confirmed the previous hypothesis derived from morphological and palynological studies, which suggested that the species belongs to *Koenigia* (Figure 5; Figure S1). Intriguingly, phylogenetic analyses revealed the unexpected finding that *K. medogensis* and *K. mollis* form a highly supported sister group (Figure 5; BS = 89%; PP = 1), despite their significant morphological differences beyond their shared characters of densely pubescent inflorescence axes and fully expanded tepals. These morphological disparities are manifested in stem morphology, indumentum types, leaf pubescence density, leaf size and shape, and other taxonomically diagnostic features in Table 1. Based on existing literature, it has been proposed that the diversification of *Koenigia* might be associated with the uplift of the Himalayas (Fan et al. 2013; Hedberg 1997). Specifically, *K. mollis* , distributed in the eastern Himalayas, is a subshrub or shrub predominantly found in temperate biomes (https://powo.science.kew.org/). In contrast, *K. medogensis* has only been discovered in Medog County, Xizang Autonomous Region. Xizang Autonomous Region is located on the southern flank of the eastern Himalayas, which has the only tropical rainforest region in China (Ding et al. 2022). Although both species are distributed within the Himalayan range, their significant differences in altitude and climatic zones suggest that they may have developed distinct ecological adaptation strategies due to geographic isolation. Furthermore, the two species are found in different habitats, with different morphology: *K. mollis* occurs in high‐altitude, cold, and arid environments by modifying leaf shape and indumentum (Table 1), while *K. medogensis* , adapted to humid, low‐light, and competitive habitats, developed asexual reproductive structures such as bulbils (Figure 3; Table 1). These morphological and ecological differences may have been shaped by geographic isolation, climatic gradients, and microhabitat adaptation following the uplift of the Himalayas.

## 5 Taxonomic Treatment

### 5.1 *K. medogensis* Bo Li, sp. nov

**FIGURE 6:**
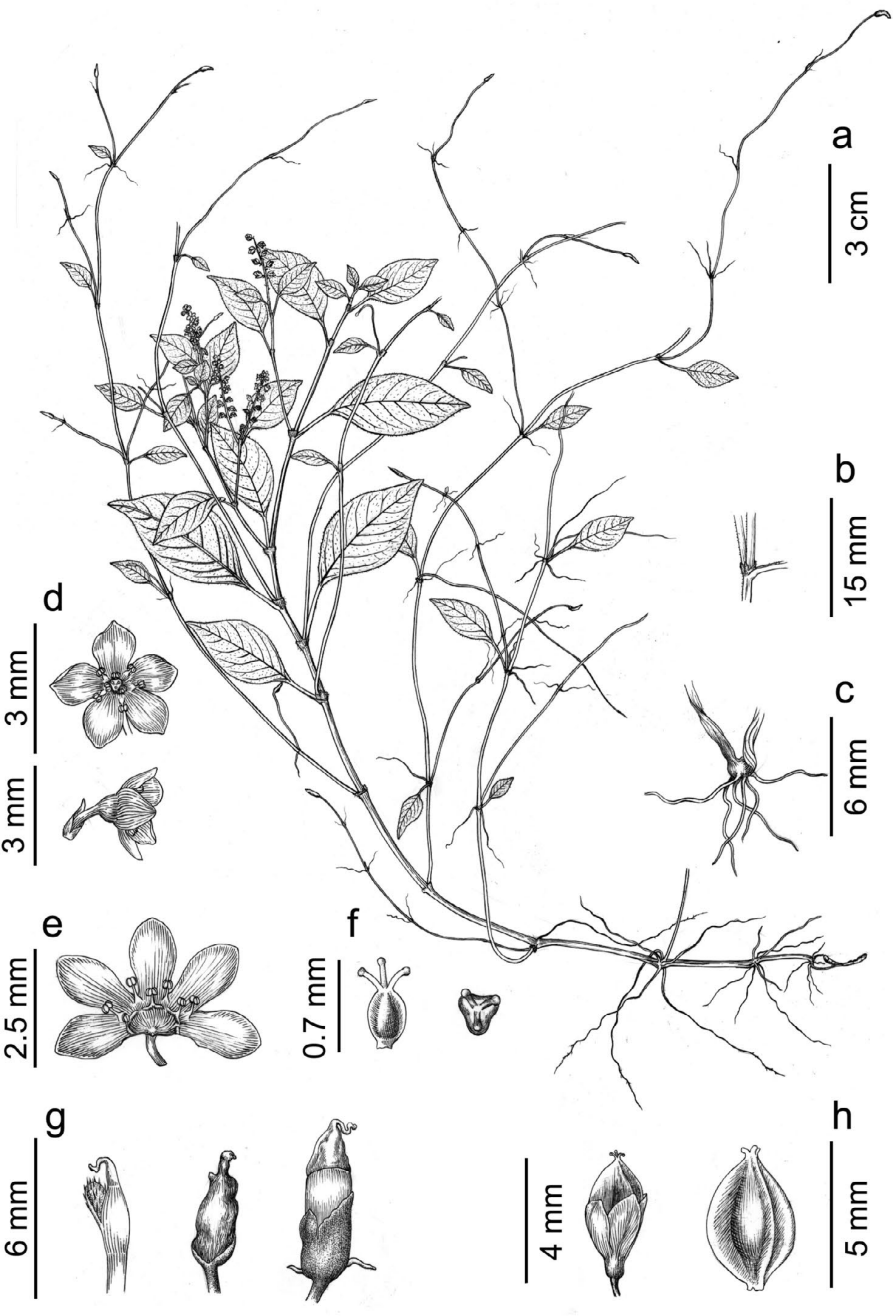
*Koenigia medogensis* Bo Li, sp. nov.: (a) habit, (b) ocrea, (c) aerial roots, (d) opened flower (frontal and lateral view), (e) dissected flower, (f) gynoecium (lateral and apical view), (g) bulbils, (h) achene in persistent perianth (left) or not (right). Drawn by Yun‐Xiao Liu from the holotype.

Figures 1, 2, 3 and 6.

#### 5.1.1 Diagnosis

*K. medogensis* and *K. mollis* both have the densely pubescent rachis and spreading flowers but differ in growth habit (perennial herb vs. semi‐shrub), reproductive mode (asexual reproduction via bulbils vs. exclusively sexual reproduction), leaf shape (ovate or ovate‐lanceolate vs. elliptic or elliptic‐lanceolate), inflorescence type (racemose and umbellate vs. paniculate), and perianth color (white or pale green vs. white).

##### 5.1.1.1 Type

CHINA. Xizang Autonomous Region: Nyingchi City, Medog County, Dexing Town, Lage Village, in wet grassy slopes, 1800 m, 29°19′42.28″ N, 95°19′51.17″ E, 24 September 2019, *Bo Li LB1043* (holotype: HITBC; isotype: CSH).

##### 5.1.1.2 Description

Herbs perennial. Stolons slender, with bulbils at tips for asexual reproduction and many aerial roots at nodes. Stems 15–50 cm tall, erect, ascending, or decumbent; slightly angular, much branched, rooting at lower nodes; sparsely pubescent or glabrous; nodes with retrorse hairs. Leaf blades ovate or ovate‐lanceolate, 1.0–3.5 × 0.5–2.2 cm, lateral veins 6–11 pairs, both surfaces sparsely pubescent. Petioles 0.5–2.3 cm, slightly winged at the base, glabrous or nearly so; base cuneate; margin entire, sparsely minutely ciliate; apex acute or shortly acuminate. Ocrea tubular, distinctly bipartite, 2.0–3.4 mm long, pale yellow‐brown to brown, membranous, slightly hyaline, base sparsely surrounded by reflexed minute hairs but glabrous above, apex acute. Inflorescences racemose, umbellate, flowers spreading, terminal or axillary; rachis densely pubescent. Pedicel short, slender, and persistent; bracts ovate, about 2.5 mm long, membranous, each 1‐flowered. Perianth white or greenish, 5‐parted; tepals broadly obovate, subequal, 1.0–2.5 mm long, apex obtuse to rounded. Stamens 8, about 0.5 mm long; anthers dark purple, ovate, distinctly separated. Styles 3, erect, very short, about 0.3 mm long; stigmas capitate. Achenes 2.0–5.0 × 1.0–3.0 mm, yellow‐brown, exceeding persistent perianth, ovoid to ovate, distinctly sharply trigonous with three prominently ridged edges and slightly concave lateral sides, surface smooth, opaque to slightly shiny.

##### 5.1.1.3 Etymology

The Specific Epithet Refers to the Type Locality, Medog County in China.

##### 5.1.1.4 Distribution, Habitat, and Phenology

*K. medogensis* can be considered endemic to Medog County as currently known. It is recorded only from two adjacent localities within Medog County (Lage Village of Dexing Town and Lagduoxiongla Valley at the border between Nyingchi County and Medog County). Both sites are situated on the eastern slopes of the Himalayas. The new species occurs in humid microhabitats, typically growing in the understory of subtropical montane dwarf forests or on moist grassy slopes at elevations of 1500–2500 m. Flowering time is June to September; fruiting time is July to October.

##### 5.1.1.5 Preliminary Conservation Status

Abundant individuals of *Koenigia medogensis can be easily found* in the above‐mentioned localities, and plenty of mature achenes can be produced in these populations. However, given the limited known distribution of the species, the lack of comprehensive studies on its area of occupancy and population size, as well as the possibility of potential threats such as human disturbance, habitat degradation, and natural disasters, we suggest evaluating the species as data deficient (DD) according to the IUCN Red List Criteria (IUCN 2024).

##### 5.1.1.6 Additional Specimens Examined (Paratypes)

CHINA. Xizang Autonomous Region: Nyingchi City, Medog County, Dexing Town, Lage Village, Duoxiongla Valley, at the edge of fir forests, 2850 m, 29°26′38.02″ N, 95°1′35.48″ E, 10 August 2024, Hu Jun 20240808B12 (CDBI).

## Author Contributions

**Xiao‐Ting Xie:** data curation (equal), investigation (equal), software (equal), writing – original draft (lead). **Yi‐Ming Wei:** formal analysis (equal), investigation (equal), writing – original draft (equal), writing – review and editing (equal). **Yong‐Jun Chen:** formal analysis (supporting), investigation (equal). **Dian‐Xiang Zhang:** supervision (equal), writing – review and editing (equal). **Jian‐Yong Shen:** formal analysis (equal), investigation (equal). **Yao‐Wu Xing:** funding acquisition (equal), resources (equal). **Bo Li:** funding acquisition (lead), investigation (supporting), project administration (lead), supervision (equal), writing – review and editing (equal).

## Conflicts of Interest

The authors declare no conflicts of interest.

## Supporting information


**Table S1:** ece372089‐sup‐0001‐AppendixS1.docx.

**Data S1:** ece372089‐sup‐0002‐DataS1.pdf.

## Data Availability

The data that supports the findings of this study is available in the Supporting Information of this article.
