# Supplementary material for: Koenigia medogensis (Polygonaceae: Persicarieae), a Distinct New Species From Xizang, Southwestern China
Source: Ecol Evol. 2025 Aug 31;15(9):e72089. doi: 10.1002/ece3.72089 (PMC12399572; doi:10.1002/ece3.72089)
Supplement: Supplementary file 1 — Table S1: ece372089‐sup‐0001‐AppendixS1.docx. [file ECE3-15-e72089-s001.docx]

## TABLE S1 | GenBank accession numbers for the taxa used in this study.

| **Taxon** | **GenBank accession numbers** | | | | | |  |
| --- | --- | --- | --- | --- | --- | --- | --- |
|  | ***matK*** | | ***rbcL*** | | ***trnL-F*** | |  |
| *Muehlenbeckia complexa* | HM851072 | | HM850184 | | JF831305 | |  |
| *Muehlenbeckia platyclada* | GQ206198 | | JN234979 | | JN235061 | |  |
| *Muehlenbeckia astonii* | ON586887 | | ON586903 | | JF831302 | |  |
| *Fallopia aubertii* | EU840492 | | EU840324 | | EU840550 | |  |
| *Fallopia dumetorum* | HM357920 | | HM357894 | | EU024785 | |  |
| *Fallopia dentatoalata* | HM357914 | | HM357888 | | EU024775 | |  |
| *Parogonum cynanchoides* | ON586884 | | ON586902 | | ON586934 | |  |
| *Pleuropterus multiflorus* | EF159150 | | HM357901 | | EU024777 | |  |
| *Reynoutria japonica* | PP066089 | | PP094362 | | JN235062 | |  |
| *Reynoutria forbesii* | ON586893 | | ON586909 | | – | |  |
| *Reynoutria sachalinensis* | ON586899 | | AF297125 | | – | |  |
| *Polygonum arenastrum* | JN895284 | | HE963604 | | KR537759 | |  |
| *Polygonum aviculare* | AB976686 | | HM850273 | | JN235060 | |  |
| *Polygonum ramosissimum* | OR823924 | | OR822966 | | KR537745 | |  |
| *Atraphaxis frutescens* | OR641027 | | KJ820721 | | OL449913 | |  |
| *Atraphaxis manshurica* | MT918050 | | JQ009268 | | JQ009286 | |  |
| *Atraphaxis spinosa* | EF437989 | | JQ009264 | | JF831296 | |  |
| *Duma coccoloboides* | JF831264 | | – | | JF831298 | |  |
| *Duma florulenta* | JF831265 | | – | | JF831299 | |  |
| *Knorringia sibirica* | EU024771 | | JN234982 | | JN235064 | |  |
| *Rheum altaicum* | EU840488 | | EU840320 | | EU840547 | |  |
| *Rheum australe* | KF906711 | | EU840309 | | AY566459 | |  |
| *Rheum compactum* | PQ428415 | | EU840319 | | AF303439 | |  |
| *Rumex acetosella* | EF438022 | | HQ590250 | | AJ583855 | |  |
| *Rumex maritimus* | JN894596 | | JN891404 | | MG780830 | |  |
| *Rumex nepalensis* | MN245083 | | JF944137 | | MK034859 | |  |
| *Oxyria digyna* | KC475052 | | KM360910 | | JN235068 | |  |
| *Oxyria sinensis* | KJ159025 | | AF297148 | | – | |  |
| *Calligonum colubrinum* | KF585081 | | KF585054 | | KF585108 | |  |
| *Calligonum ebinuricum* | JQ731650 | | JQ731655 | | JX987213 | |  |
| *Calligonum pumilum* | JX259349 | | JX259334 | | JX259363 | |  |
| *Calligonum roborowskii* | JX259347 | | JQ009270 | | JQ009288 | |  |
| *Pteroxygonum denticulatum* | HM357915 | | HM357889 | | HQ843149 | |  |
| *Pteroxygonum giraldii* | GU373528 | | GQ206230 | | EU402464 | |  |
| *Fagopyrum dibotrys* | GQ434135 | | JN234983 | | JN235065 | |  |
| *Fagopyrum esculentum* | JF829981 | | JN187116 | | EU024792 | |  |
| *Fagopyrum tataricum* | JF829984 | | D86287 | | HQ843172 | |  |
| *Fagopyrum leptopodum* | JF829978 | | AB000313 | | PQ497588 | |  |
| *Koenigia alpina* | HM357917 | | FM883602 | | KR537738 | |  |
| *Koenigia forrestii* | AY042605 | | AF297144 | | JN235043 | |  |
| *Koenigia hookeri* | KT280246 | | EU840289 | | EU840541 | |  |
| **TABLE S1 \|** (continued) | |  | |  | |  | |
| **Taxon** | | **GenBank accession numbers** | | | | | |
|  |  | ***matK*** | | ***rbcL*** | | ***trnL-F*** | |
| *Koenigia islandica* | EU840455 | | EU840287 | | KF541257 | |  |
| *Koenigia mollis* | GQ206190 | | EF653764 | | EF653790 | |  |
| *Koenigia polystachya* | MF786824 | | MF786529 | | JN235040 | |  |
| *Koenigia nummulariifolia* | OM638596 | | JN234961 | | JQ360845 | |  |
| *Koenigia tortuosa* | MF786792 | | HQ435357 | | JN235038 | |  |
| *Koenigia campanulata* | MH324415 | | JN234953 | | KR537755 | |  |
| *Koenigia songarica* | MT918087 | | MT931217 | | EU024788 | |  |
| *Koenigia medogensis* | MZ061715 | | MZ061718 | | MZ054186 | |  |
| *Bistorta amplexicaulis* | EF438014 | | JN234952 | | JN235033 | |  |
| *Bistorta officinalis* | AF204859 | | FM883607 | | KR537728 | |  |
| *Bistorta suffulta* | MH659519 | | JN234990 | | JN235073 | |  |
| *Persicaria hydropiper* | HM357924 | | EF653780 | | JN235045 | |  |
| *Persicaria perfoliata* | HM357916 | | HM357890 | | FJ627264 | |  |
| *Persicaria posumbu* | GU266606 | | EF653778 | | JN235044 | |  |
| *Persicaria viscosa* | EU196965 | | HQ435349 | | EU197051 | |  |
| *Oxygonum sinuatum* | KR734898 | | KR736460 | | KR537752 | |  |
| *Oxygonum salicifolium* | – | | – | | KR537750 | |  |
| *Oxygonum magdalenae* | – | | – | | KR537737 | |  |
| *Antigonon leptopus* | KR537868 | | AF297146 | | MG913344 | |  |

## TABLE S2 | Dataset characteristics used for phylogenetic analyses.

| **Dataset** | **Number of taxa** | **Length**  **(bp)** | **Variable sites** | **Parsimony informative sites** | **Proportion of parsimony informative sites** |
| --- | --- | --- | --- | --- | --- |
| *matK* | 58 | 2670 | 1245 | 613 | 22.96 % |
| *rbcL* | 56 | 2961 | 954 | 661 | 22.32 % |
| *trnL*-*F* | 57 | 1513 | 739 | 519 | 34.30 % |
| combined cpDNA | 60 | 7144 | 2938 | 1793 | 25.10 % |

| 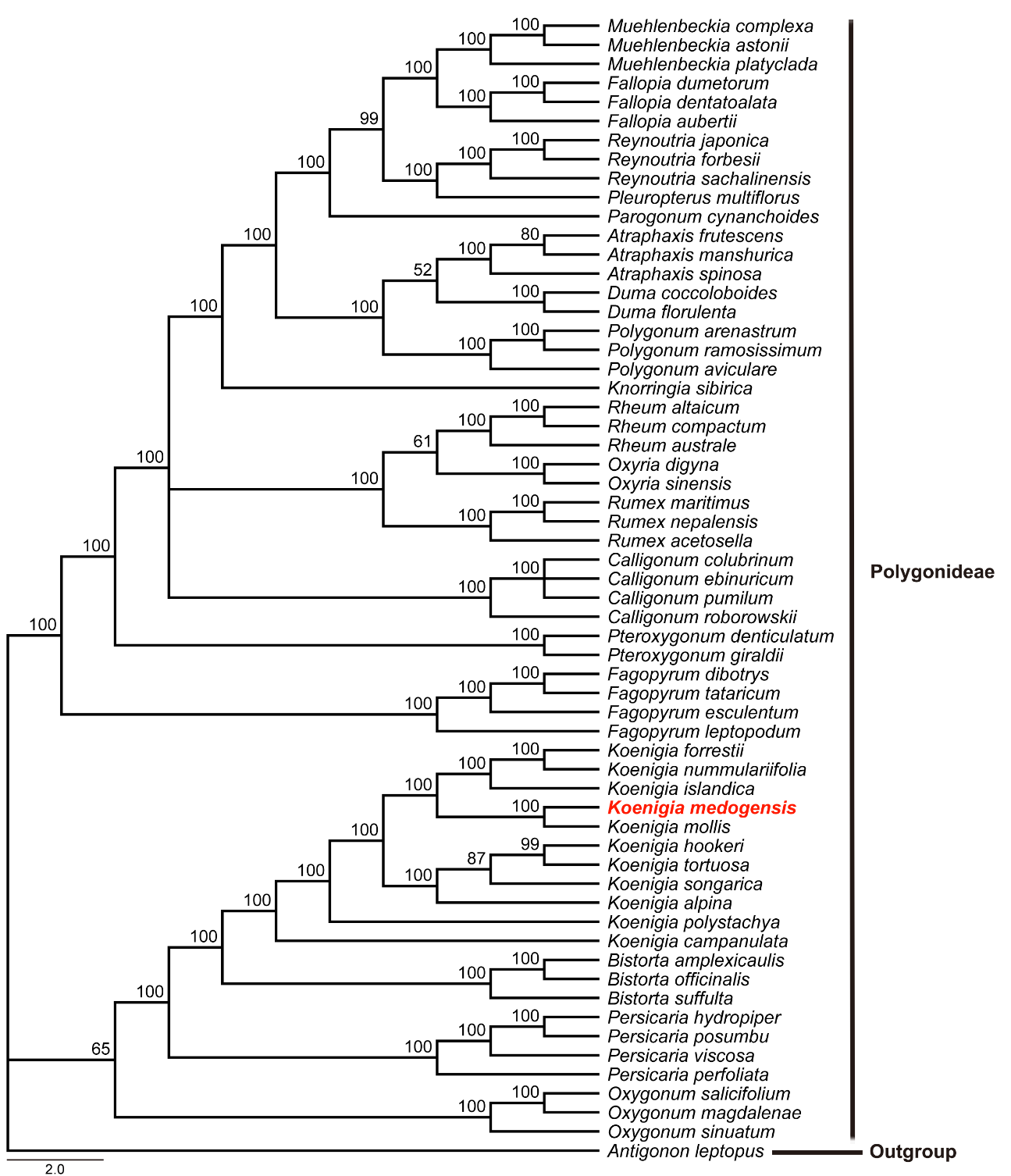 |
| --- |
| **Figure S1.** Bayesian phylogenetic tree based on of the cpDNA (*matK*, *rbcL*, and *trnL-F*) dataset of the 60 accessions representing 19 genera of Polygonoideae. Numbers above branches indicated Bayesian posterior probabilities (PP). New species are shown in red and bold. |
